# Vulvovaginal Collagen Injection as a Regenerative Strategy in Genitourinary Syndrome of Menopause: Results of a Pilot Study

**DOI:** 10.3390/jcm15041408

**Published:** 2026-02-11

**Authors:** Ana Isabel Borobia Pérez, Javier Jesús Estévez Espejo, María Jiménez-González, Roger David García López

**Affiliations:** 1 Department of Physical Medicine and Rehabilitation, La Paz University Hospital, Paseo de la Castellana 261, Fuencarral-El Pardo, 28046 Madrid, Spain; javierjesus.estevez@salud.madrid.org (J.J.E.E.); rogerdavid.garcia@salud.madrid.org (R.D.G.L.); 2 Bioestadística–Plataforma de Bioestadística y Bioinformática Instituto de Investigación Hospital La Paz, La Paz University Hospital, Paseo de la Castellana 261, Fuencarral-El Pardo, 28046 Madrid, Spain; jimenezglezmaria@gmail.com

**Keywords:** vulvovaginal, genitourinary syndrome of menopause, collagen injection, conservative therapies, regenerative medicine, retrospective study, VAS

## Abstract

**Background/Objectives:** Genitourinary syndrome of menopause (GSM) is a common and under-diagnosed condition that significantly affects the quality of life of post-menopausal women. Conventional treatments, especially those based on estrogens, have limitations, which has prompted the search for alternative therapies in the field of regenerative medicine. In this context, intradermal injectable collagen, with regenerative and analgesic properties, could represent an innovative option. This study aims to evaluate the efficacy and safety of multipoint intradermal injections of collagen (MD-Tissue Guna SpA, Milan, Italy) in the treatment of GSM refractory to conventional first-line therapy. **Methods**: A retrospective study was conducted in 20 patients diagnosed with GSM. Intradermal injections of collagen were administered in the vulvovaginal region. Clinical outcome was assessed using the Visual Analog Scale (VAS) for baseline pain and dyspareunia, the Vaginal Health Index (VHI), the Vulvar Health Index (vHI), the Vulvovaginal Symptoms Questionnaire (VSQ) and the Clinical Global Impression scale (CGI). **Results**: Three months after end of injections, baseline pain measured using the VAS was significantly reduced from a mean of 5.9 to 0.8 (*p* < 0.001), and pain during intercourse decreased from 8.7 to 2.0 (*p* < 0.001). Significant improvements were also observed in the tissue parameters of VHI and vHI. In the VSQ, 100% of patients reported improvement in at least one domain, including itching, dryness, burning, and social and sexual impact. According to the CGI scale, 80% reported feeling ‘much better’ and 20% ‘moderately better’. No relevant adverse effects were reported. **Conclusions**: Intradermal vulvovaginal collagen injections appear to be a safe and potentially effective intervention in improving pain and other symptoms of GSM, within the regenerative medicine approach. These preliminary results justify prospective studies, with larger sample sizes and long-term follow-up, to confirm and consolidate clinical utility.

## 1. Introduction

### 1.1. Background

Genitourinary syndrome of menopause (GSM), proposed in 2014 by the North American Menopause Society, replaces terms such as vulvovaginal atrophy or atrophic vaginitis to more broadly encompass the set of signs and symptoms resulting from the decline in estrogen and androgens after menopause. This syndrome affects both the vulvovaginal area and the lower urinary tract, and it commonly manifests with dysuria, urinary urgency, recurrent urinary tract infections and incontinence. Vulvovaginal symptoms, however, such as vaginal dryness (55–81%), dyspareunia (44–74%), sexual dysfunction (55%) and vulvar irritation, itching, pruritus or ecchymosis (37%), are the most frequent and disabling. Dyspareunia, in particular, is reported as the most bothersome symptom by most patients. These alterations negatively impact the quality of life of up to 80% of affected women and their sex life in 75% of cases (Table 1) [1,2,3,4,5,6,7,8]. Histologically, GSM is associated with thinning of the vulvovaginal epithelium and mucosa, reduced vascularization, loss of nerve endings, and reduced vaginal secretion. There is also less glycogen synthesis and a decrease in elastin and collagen fibers. This last change, particularly relevant in our study, implies a decrease in remodeling and an increase in collagen degradation. This can lead to an overall loss of up to 75% of type I collagen in various connective tissues, such as skin, subcutaneous tissue, fasciae, ligaments, muscles or trabecular bone. In the genitourinary system, this collagen deficit compromises the structural support, mechanical properties and function of the connective tissue in the structures of the pelvic floor. This has negative repercussions on vulvovaginal turgor and tissue resistance of the epithelium. In short, this structural deficit aggravates the symptoms and favors progression of the [5,9] syndrome. The prevalence of GSM is high. It is estimated that 50–70% of post-menopausal women will experience some of its symptoms in their lifetime [10]. In Spain, the multi-center GENISSE study confirmed a prevalence of 70% in women attending gynecological consultations [11]. Despite this, however, GSM remains under-diagnosed and under-treated, particularly with regard to vulvovaginal symptoms. Fewer than 30% of women seek medical attention for these symptoms, and more than 70% do not verbalize them because they are embarrassed or consider them a ‘normal’ part of aging [1,5,12,13]. Meanwhile, the progressive aging of the population suggests that its incidence will continue to increase in the coming decades. GSM diagnosis is clinical and requires a detailed medical history (gynecological, sexual, prior treatment, irritating factors), an exhaustive physical examination, and specific complementary questions (Vaginal Health Index, Vulvar Health Index, Vulvovaginal Symptoms Questionnaire). Although in most cases no additional tests are required, cytology, culture, or biopsy may be indicated in doubtful situations or when differential diagnoses with infections, vulvar dermatoses, vulvodynia, neoplastic lesions, or pelvic floor dysfunction are raised [1,4,14]. Current treatment of genitourinary syndrome of menopause (GSM) is based on a stepwise approach combining hygiene and dietary measures [5,7,8], physical and rehabilitative therapy (pelvic floor exercises, physiotherapy, manual techniques and regular physical activity [5,7,8]), symptomatic therapy, hormonal treatment and, more recently, regenerative therapies. Vaginal lubricants and moisturizers are the first line, being useful for mild symptoms or for women unwilling or unable to receive hormones, although they do not reverse the underlying pathophysiology [5,7,8,15]. In moderate-to-severe cases, topical estrogens remain the first-line therapeutic mainstay, with proven efficacy on vaginal and urinary symptoms and a superior safety profile to systemic treatment [5,7,8,12]. Alternatives such as intravaginal dehydroepiandrosterone (DHEA) or oral ospemifene have been shown to improve sexual function and vaginal trophism, even in women with a history of breast cancer after completion of adjuvant treatment [5,7,8,16]. Low adherence, side effects, and contraindications in women with a history of hormone-dependent cancer, however, limit their use [7]. This has favored the rise of non-hormonal therapies within regenerative medicine. Among these, hyaluronic acid, both topical and injectable, has been shown to improve hydration, tissue elasticity, and vaginal symptoms, with a favorable safety profile [17,18]. Fractional CO_2_ laser has also shown positive results in improving sexual function and vulvovaginal symptoms, although more robust studies are still required to confirm its long-term efficacy [19,20,21]. Another alternative is radiofrequency, which promotes neo-collagenases and neo-vascularization, restoring vaginal mucosal elasticity and reducing symptoms of GSM [22,23]. In parallel, platelet-rich plasma (PRP) provides growth factors that stimulate tissue regeneration, and comparable improvements to local hormonal therapy have been reported in reducing dryness and dyspareunia [24,25]. Finally, in oncology patients, where hormonal therapy is contraindicated, several reviews highlight the growing role of these non-hormonal strategies as safe and effective alternatives for GSM management [26]. These limitations of standard treatments and the need for safe approaches in vulnerable populations, such as cancer survivors [26], reinforce the interest in new regenerative strategies such as injectable collagen, which could represent an innovative option in this scenario. Among these emerging options, injectable type I collagen (MD-Tissue, GUNA Spa, Milan, Italy) has recently emerged as an innovative alternative in regenerative medicine. It has been investigated in different clinical contexts, including a randomized trial in plantar fasciitis, a pilot study in lumbar spondylosis, and a pilot randomized controlled trial combining in situ collagen injection with rehabilitative treatment in long-lasting facial nerve palsy, reporting improvements in pain and/or functional outcomes [27,28,29]. Regarding its mechanism of action, preclinical evidence suggests that this collagen-based medical device may act as a mechanical scaffold influencing morpho-functional properties of cultured human tenocytes [30]. Although the in vivo biological pathways are not fully elucidated, this scaffold-based effect supports the hypothesis that collagen injection could contribute to tissue support and repair, potentially improving vulvovaginal mucosal turgor, hydration, and resilience—key features involved in GSM pathophysiology [1,5,31,32,33]. Unlike symptomatic treatments, injectable collagen acts at the tissue level, aiming to promote structural regeneration. Collagen injections have been explored in pelvic pain related to post-partum episiotomy and cesarean scars [34]. Periurethral collagen injections have also been used as bulking therapy for stress urinary incontinence [35,36]. However, clinical evidence supporting intradermal vulvovaginal collagen specifically for GSM remains limited.

### 1.2. Rationale of the Study

Based on our knowledge of the pathophysiological mechanisms of GSM and the biostimulatory effect of injectable collagen MD-Tissue, we propose to evaluate the effect that vulvovaginal intradermal injections may have in contributing to the regeneration of connective and epithelial tissue, reducing clinical symptoms, and improving quality of life in post-menopausal women. The ability of collagen to modulate fibroblast activity, induce endogenous collagen synthesis, and participate in the reorganization of the extracellular matrix positions it as a promising candidate for regenerative therapies of the pelvic floor.

### 1.3. Aim of the Study

The study set itself one primary objective and three secondary objectives.

(1) Primary aim: The primary aim of this study was to examine the efficacy of multipoint intradermal injections of collagen (MD-Tissue) in the vulvovaginal area in patients with GSM refractory to conventional first-line therapy. The main criterion for efficacy was considered to be pain reduction, as assessed by the Visual Analog Scale (VAS).
(2) Secondary aims:
  - Analyze the improvement in symptomatology associated with GSM as measured by validated questionnaires.
  - Analyze impact on general and sexual quality of life using validated questionnaires. Assess patient satisfaction with the treatment received using the specific scale.

## 2. Materials and Methods

### 2.1. Type of the Study

A retrospective follow-up observational study involving medicinal products, conducted between 2022 and 2025 with patients with the main diagnosis of GSM assessed in the Pelvic Floor Rehabilitation Unit. Data collection from medical records took place between 27 February 2025 and 30 June 2025. This study was approved by the medicine Research Ethics Committee (REC) of La Paz University Hospital accredited by the Spanish Ministry of Health and Consumer Affairs and by the health authorities of the Autonomous Community of Madrid, https://www.aemps.gob.es/medicamentos-de-uso-humano/investigacion_medicamentos/investigacionclinica_ceim/ (accessed on 27 February 2025).

A concurrent control group was not included because this work was conceived as a single-arm retrospective pilot study, based on consecutive patients treated in routine clinical practice. At the time of data collection, there was no standardized “usual care” comparator with the same follow-up schedule and the same set of validated PROMs applied systematically in our unit. Additionally, participants had heterogeneous prior therapies and/or contraindications or refractoriness to first-line options. Therefore, a within-subject pre-post design was selected to assess feasibility and safety, as well as to obtain preliminary effect size estimates to inform the design and sample size calculation of a future controlled trial.

### 2.2. Study Population

The study population consisted of women under follow-up at the Pelvic Floor Rehabilitation Clinic with a diagnosis of genitourinary syndrome of menopause (GSM). The diagnosis was made by a specialist in Physical and Rehabilitation Medicine through detailed anamnesis, specific pelvic floor physical examination, and the use of validated clinical questionnaires. Other potential causes of pelvic pain were ruled out through clinical evaluation, medical history review, and complementary tests when necessary. All patients included received intradermal injection of MD-Tissue collagen and met the following inclusion and exclusion criteria.

- Inclusion criteria:
  - Over 18 years of age.
  - Genitourinary syndrome of menopause.
  - No association with other pelvic pain conditions.
  - Refractory to conventional first-line treatments listed in clinical practice guidelines.

Capacity to understand and sign the informed consent for the MD-Tissue collagen injection procedure, in accordance with standard clinical practice.

- Exclusion criteria:
  - Mental or cognitive disorder that impedes comprehension.
  - Local infections at the site of injection.
  - Total or partial denervation of the pelvic floor.
  - Neurological diseases: CVA, LM, MS.
  - Active urinary tract infections and vulvovaginosis.
  - Lichen sclerosus.

### 2.3. Assessment Variables

#### 2.3.1. Primary Variable

Reduction of pain caused by GSM symptoms by injection with MD-Tissue collagen, using the Visual Analog Scale (VAS) (Figure S1). We assessed pain in two circumstances: pain during sexual intercourse and the patient’s ongoing baseline pain.

#### 2.3.2. Secondary Variables

- Modified Vaginal Health Index (VHI) (Table S1).
- Vulvar Health Index (vHI) (Table S2).
- Vulvovaginal Symptoms Questionnaire (VSQ) (Table S3).
- Clinical Global Impression Scale (CGI) (Table S4).

#### 2.3.3. Demographic and Other Clinical Variables

The following data were also collected: age, reason for referral, main diagnosis, associated diagnoses, and previous treatments, specifying the types of therapies used.

In addition, the possible adverse effects of collagen injections were collected.

### 2.4. Data Collection and Study Protocol

Data were collected from the medical records of all patients for the diagnosis of genitourinary syndrome of menopause. Initially, these patients were referred to the pelvic floor rehabilitation clinic for chronic pelvic pain. After an exhaustive medical history and physical examination, a diagnosis of GSM was made. For treatment, they were initially treated, following clinical practice guidelines, with conventional first-line treatments (lubricants, moisturizing creams and topical estrogen). After their symptoms proved refractory to these treatments, intradermal injections of MD-Tissue collagen were started.

The collagen injection treatment protocol consisted of three initial sessions, each spaced four weeks apart, followed by a clinical check-up one month after the third session. If symptoms persisted at that point, a fourth and fifth injection were administered, also four weeks apart, maintaining the consistency of the treatment interval. This approach was based on our clinical practice, as there are currently no published protocols regarding the use of injectable collagen for genitourinary syndrome of menopause (GSM). Our protocol was designed by adapting the therapeutic schemes used in musculoskeletal applications of collagen and from existing guidelines for platelet-rich plasma (PRP) in the treatment of vulvovaginal atrophy. This allowed us to preserve the sequence and therapeutic rhythm between sessions while tailoring treatment to the patient’s individual response.

All study variables were collected from the medical records before the first collagen injection (pre-injection data) and three months after the last collagen injection (post-injection data). RedCap software (version 10.3.1, Nashville, TN, USA) was used for data collection.

Subsequently, a descriptive study of the demographic and clinical variables was performed, as was a comparative study of the rest of the variables. The comparative statistical analysis of the pre- and post-injection data was conducted with the GraphPad Prism (version 10.6, San Diego, CA, USA).

### 2.5. Injection Technique

The technique used for injection was a multipoint technique infiltrating intradermally over the vulvar area, over the labia minora and fourchette, and the posterior vaginal wall. It infiltrates at an average of 0.1–0.2 mL/point, leaving a space of 0.5 cm between injection sites, up to a maximum of 2 mL, which is the content of a vial of MD-Tissue collagen, and uses a 30 G-gauge needle. All this was implemented with the patient in lithotomy position under complete aseptic measures and prior analgesia with topical lidocaine.

### 2.6. Safety Assessment Criteria

Safety data were collected through active surveillance at each follow-up visit, which occurred monthly during the treatment and at the three-month post-treatment visit. A standardized checklist was used during consultations to systematically evaluate and record any potential adverse events. Local adverse events monitored included injection site pain, bleeding, bruising, infection, nodules, scarring, and signs of allergic reaction. All data were extracted from electronic medical records and cross-verified with physician notes and nursing records.

### 2.7. Questionnaires Characteristics

For the diagnosis and assessment of GSM, the following validated questionnaires and clinical scales were used, in addition to the medical history and physical examination in routine clinical practice:

- The Visual Analog Scale (VAS) was used to measure the intensity of pain perceived by patients, both at rest and during sexual relations (SR). It consists of a 10 cm line where 0 represents ‘no pain’ and 10 represents ‘the worst pain imaginable’. Patients marked the point that reflected the intensity of their pain (Figure S1).
- The modified Vaginal Health Index (VHI) comprises four items (elasticity, discharge volume, epithelial integrity, and moisture). Each symptom is classified from 1 to 5, with 1 being the worst situation and 5 the best. GSM is defined when a total score below 12 is identified at the beginning of the study (Table S1).
- The vulvar health index (vHI) consists of five items (labia majora and labia minora, clitoris, vestibule and elasticity, color, discomfort and pain). Each item is graded with a score ranging from 0 (best clinical condition) to 3 (worst clinical condition). GSM is graded into mild (0–5 points), moderate (6–10 points) and severe (>10) (Table S2).
- To assess the impact on quality of life and sex life, the vulvovaginal symptoms questionnaire (VSQ) was used, which consists of 21 items (itching, burning or stinging, pain, irritation, dryness, discharge, vulvar or vaginal odor, worry about vulvar symptoms, appearance, frustration about symptoms, embarrassment about symptoms, effects of symptoms on social interactions and desire to be with others, symptoms making it difficult to show affection, affecting daily activities, affecting desire for intimacy, whether they are sexually active with a partner, vulvar symptoms having an effect on sexual relations, causing pain, dryness or bleeding during intercourse). Each item has only two possible responses to be checked by the patient, ‘Yes’ and ‘No’ (Table S3).
- To assess patient satisfaction with treatment, we use the Clinical Global Impression (CGI) scale. It provides a measure of the overall change in clinical status from baseline to the time of assessment. This scale aims to reflect the perception of change experienced by the patient, considering the improvement or worsening of symptoms. Scores range from 1 (much better) to 7 (much worse) (Table S4).
- Finally, patients were questioned and the expected side effects, such as persistent edema (>24 h), ecchymosis, necrosis, and infection after injection, were discussed.

Data from these questionnaires were collected from the medical appointment before starting treatment with collagen injections and three months after the last injection was completed, as shown in the flowchart.

### 2.8. Statistical Analysis

The data acquired were analyzed using the GraphPad Prism (Version 10; San Diego, CA, USA). These will be shown as absolute and relative frequencies in the case of qualitative variables, and with the main measures of dispersion (mean, standard deviation, median and interquartile range) in the case of quantitative variables. The Kolmogorov–Smirnov test was performed to study the normality of the variables, assuming normality for all continuous variables. For the analysis of the main aim, the Pearson Chi-Square test (or Fisher’s exact test for 2 × 2 tables or likelihood ratio in tables, if necessary) was used for qualitative variables, and Student’s *t*-test for repeated measures for quantitative variables. The significance level was set at <0.05.

### 2.9. Ethical Considerations

The study was conducted in accordance with the principles of the Declaration of Helsinki and current data protection regulations (General Data Protection Regulation and Spanish Organic Law 3/2018). As a retrospective and observational study, all data were anonymized prior to analysis.

The study protocol was reviewed and approved by the Ethics Committee for Research with Medicines (CEIm) of Hospital Universitario La Paz, accredited by the Spanish Ministry of Health, and by the health authorities of the Community of Madrid.

Given the retrospective nature of the study and the exclusive use of anonymized data from electronic medical records, individual informed consent was not required, in accordance with the ethics committee’s approval.

## 3. Results

### 3.1. Patients Included

Twenty patients met the inclusion and exclusion criteria and were included in this retrospective clinical evaluation study after receiving specific interventional treatment. No records were excluded, and all included cases were validated for analysis.

All patients ultimately received a total of five collagen injection sessions, as their symptoms persisted beyond the third application. This consistent therapeutic exposure helped ensure homogeneity in the treatment approach across the sample, thus avoiding the need for subgroup or sensitivity analyses based on the number of sessions.

### 3.2. Qualitative Analyses

In the exploratory analysis phase, demographic, clinical, and therapeutic history variables were characterized. The baseline characteristics of the study population are presented in Table 2. The median age was 54 years (range: 48–58), with a homogeneous distribution of diagnoses. Pelvic pain was the most frequent reason for referral (55%), followed by urinary incontinence (30%), vulvodynia (10%) and dyspareunia (5.0%). All patients had a confirmed diagnosis of GSM in rehabilitation consultation (Table 2).

GSM in 100% of patients was initially treated conservatively. Some patients had received multiple lines of therapy prior to joining the study. The most frequent treatments included the following: lubricants, used by 55% of patients; moisturizers with hyaluronic acid, used by 50%; moisturizers without hyaluronic acid, used by 25%; local and systemic hormonal therapies, used by 20% and 5%, respectively; cream with lidocaine, used by 5%; and ospemifene, used by 5% (Table 3).

In addition, the most frequently occurring symptoms and signs in patients were documented. Irritation/itching/burning occurred in 100% of patients, vaginal dryness in 90%, dyspareunia/sexual dysfunction in 75%, urinary incontinence in 45% and over-active bladder in 5% (Table 4).

### 3.3. Primary Study Variable: Pain Reduction Through VAS

**Figure 1:**
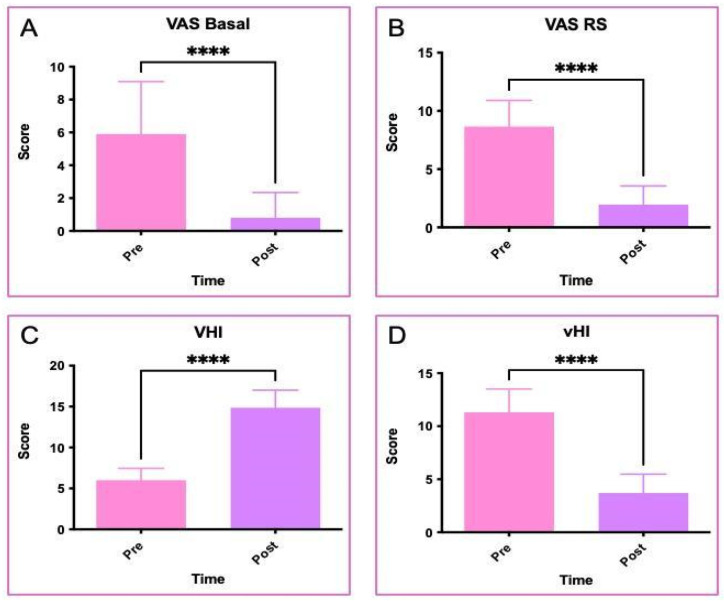
VAS, VHI and vHI analyses. (**A**) VAS at basal level; (**B**) VAS at sexual intercourse; (**C**) VHI; (**D**) vHI. Statistical differences obtained by Student’s *t*-test; **** *p* < 0.0001; pink color: pre-treatment status, violet color: post treatment.

Three months after the last collagen injection, pain measured using the Visual Analogue Scale (VAS) showed a statistically significant decrease. Baseline pain by VAS (VAS Basal), score in pre-treatment setting was equal to 5.90 ± 3.19, while the mean VAS value to a mean of 00.80 post-treatment setting was equal to 0.80 ± 1.54 (*p*-value < 0.0001; Figure 1A). Indeed, the VAS during sexual intercourse (VAS RS) was also reduced from a mean of 8.65 ± 2.25 before treatment to a mean of 1.95 ± 1.60 after treatment, showing a mean reduction of 6.70 points on the VAS (*p*-value < 0.0001; Figure 1).

### 3.4. Secondary Study Variables: VHI and vHI

A significant increasement in Vaginal Health Index (VHI; Figure 1) values was identified from a mean of 6.00 ± 1.45 points before treatment, which corresponds to a diagnostic score of GSM, to a mean of 14.85 ± 2.16 points after treatment, a score higher than the value defining a diagnosis of GSM (*p*-value < 0.0001; Figure 1C). Similarly, the Vulvar Health Index (vHI) showed a statistically significant reduction from a mean of 11.30 ± 2.20 points before treatment, corresponding to a severe grade of GSM, to a mean of 3.70 ± 1.78 points after treatment, corresponding to a mild grade of GSM (*p*-value < 0.0001; Figure 1D).

### 3.5. Vulvovaginal Symptoms Questionnaire (VSQ)

In addition, 21 clinical items from the Vulvovaginal Symptoms Questionnaire (VSQ) were analyzed and statistically significant improvements were observed between pre- and post-treatment conditions (Table 5). In terms of parameters related purely to GSM symptomatology, 100% of patients reported improvement in itching, burning or stinging sensation and vulvar irritation, 95% reported no vaginal or vulvar dryness after treatment, and 80% reported no vulvar pain. In relation to the parameters closely associated with the psychosocial sphere, after treatment, 100% of patients reported not feeling frustrated by their symptoms, not feeling ashamed of them, symptoms not affecting their social relationships, and not preventing them from showing affection to other people (Table 5). Only 5% reported being worried about the appearance of their vulva or worried about their vulvar symptoms at the end of treatment. In the sexual sphere, 100% of patients regained the desire for intimacy and all of them experienced a complete improvement in dryness and bleeding during intercourse (Table 5). Approximately 80% of patients reported feeling much better and 20% felt moderately better according to the Clinical Global Impression scale (Table 6). Finally, no adverse events were reported in any of the patients. All follow-up visits included active monitoring for local and systemic side effects, and none of the evaluated complications—such as pain, bleeding, bruising, infection, nodules, or allergic reaction—were documented.

## 4. Discussion

Genitourinary syndrome of menopause (GSM) is a condition that significantly affects the biopsychosocial, occupational, and sexual domains of women. This impact highlights the need for a more proactive diagnostic approach, allowing earlier and more accurate identification and implementation of effective treatments, mainly focused on pain reduction (the main aim of our study) and alleviation of associated symptoms and signs. In our sample, it was surprising that none of the patients referred to the rehabilitation clinic had a prior diagnosis of GSM. Approximately 55% of referrals were due to pelvic pain, 30% to urinary incontinence, 10% to vulvodynia and 5% to dyspareunia, without considering GSM as a potential diagnosis. Following an adequate medical history and physical examination in the rehabilitation consultation, a diagnosis of GSM was established in 100% of the cases. This finding underlines the frequent under-diagnosis of this pathology, as documented in previous studies, such as the one by E. Moral et al. [11], in which GSM was diagnosed in 60.2% of women with no known prior diagnosis [1,5,11]. GSM has a considerable impact on women’s quality of life, not only because of its physical symptoms, but also because of its effects on psychological and sexual health. It is essential that health professionals are trained to identify and properly diagnose this condition and can guide patients through the different treatment options available. Following current clinical guidelines, patients in our study were initially given conventional first-line treatment, including vaginal lubricants, moisturizers and topical estrogens [2,3,5]. Approximately 55% of patients used vaginal lubricants, 50% used moisturizers with hyaluronic acid, 25% used moisturizers without hyaluronic acid, 20% received local estrogen treatment, 5% systemic estrogen treatment, and a small percentage used other treatments such as ospemifene and lidocaine cream. No significant improvement was observed with these therapies, prompting the transition to a second-line treatment: intradermal collagen injections. The lack of response to conventional treatments can be explained by a number of factors. According to the REVIVE study [7], nearly two out of five women drop out of treatment due to dissatisfaction with results, low tolerability, or discomfort with product use. Thirty-six percent of participants dropped out due to discomfort with daily or frequent application, discomfort with product texture, odor or residue, and fears related to systemic adverse effects, such as breast cancer risk. These fears persist even when locally acting treatments are used. In addition, many women reported not having received an adequate explanation of how to use the product or what to expect, and lack of professional follow-up negatively affected adherence. In our sample, only 5% accepted systemic hormonal treatment because of fears related to possible adverse effects, such as breast cancer [5]. These findings reinforce the need to explore alternative therapies, such as regenerative treatments with injectable collagen. These therapies offer potential benefits such as longer duration of effect, structural tissue improvement (beyond symptomatic relief), a favorable safety profile, fewer applications, and better overall tolerability. In our study, positive clinical effects were observed in these areas. Although there are some studies emerging that back up the use of different regenerative techniques in pelvic floor pathologies, including GSM [5,19,20,21,22,23,24,25,26], no previous literature has been found documenting the specific use of injectable collagen for this particular indication. The main aim of our study was to evaluate the efficacy of collagen injections in the treatment of GSM, considering as a measure of efficacy the significant reduction in baseline pain and pain during intercourse, both assessed using the Visual Analog Scale (VAS). The pain is the predominant symptom in GSM [5,10], as reflected in our sample, where 100% of patients experienced continuous burning and 75% had dyspareunia, defined as persistent genital pain before, during or after intercourse [37] Three months after the last injection, a statistically significant mean reduction of 5.10 points in baseline pain was observed, implying an 86.44% decrease in baseline pain intensity and, in some cases, complete elimination of pain. In addition, there was a statistically significant mean decrease of 6.7 points in pain during sexual relations (SR), meaning a 77.01% reduction in pain intensity in SR. These results show promising pain reduction outcomes following collagen injection in GSM and may compare favorably with those previously reported in the literature; however, direct comparisons should be made with caution due to differences in study design, populations, and evaluation criteria. Although there are studies showing promising results in this field, the scientific evidence is still limited. The only relevant precedent is the randomized pilot clinical trial conducted by Romero-Cullerés et al. [34] in the context of post-episiotomy perineal scars. In the study, a significant reduction in pain was observed in the intervention group, especially on the McGill sensory sub-scale (−15.1 vs. −6; *p* = 0.040). Likewise, the Visual Analog Scale (VAS) in the intervention group (collagen) was reduced by an average of 3.6 points, initially going from 5.7 points to 2.1 points after injection (*p* < 0.05) [34]. This reduction objectively quantifies the analgesic effect of injectable collagen treatment. On the other hand, the successful use of injectable collagen has been documented, also in terms of pain improvement measured using the VAS, in pathologies of other locations but with similar pathophysiological mechanisms of deficit, damage, or natural degeneration of the connective tissue. These include rotator cuff tendinopathy, epicondylitis, osteoarthrosis, plantar fasciitis, facial paralysis and myofascial syndrome (trapezius, masseter, pyramidal), with positive results in terms of pain and quality of life [27,28,29,30]. Clinically, these effects translate into an increase in the turgor and consistency of the epithelium, observable improvement in hydration, reduction in erythema and visible atrophy, as well as progressive recovery of elasticity and functional sensitivity of the treated area. In addition, increased resistance to local microtrauma and an overall improvement in vulvovaginal appearance and function have been reported, particularly relevant in the context of genitourinary syndrome of menopause (GSM) [38]. In our study, these effects were reflected in both clinical improvement of symptoms and tissue recovery as assessed by two validated scales: the modified Vaginal Health Index (VHI) and the Vulvar Health Index (vHI). Both scales are validated for the clinical assessment of genitourinary syndrome of menopause and are widely used in clinical practice and research trials, as they allow objective quantification of key aspects of genital trophism such as hydration, elasticity, tenderness, color and epithelial integrity. Their application facilitates not only diagnosis, but also monitoring of clinical course and response to treatment in women with GSM [5]. In relation to the modified Vaginal Health Index (VHI), a significant improvement was observed after treatment, from a baseline mean of 6.0 points (6.0–6.0) before treatment, indicative of a diagnosis of GSM, to 14.9 points (13.5–16.0) after the intervention, far exceeding the diagnostic threshold for the syndrome (*p* < 0.001). This increase reflects an objective recovery of vaginal health, with substantial improvements in elasticity, epithelial integrity, discharge volume and moisture, all key indicators of GSM vaginal trophism. Likewise, the Vulvar Health Index (vHI) showed a clinically relevant and statistically significant improvement, decreasing from a mean of 11.3 points (10.5–13.0) before treatment, indicative of severe GSM, to 3.7 points (3.0–5.0) after the intervention, corresponding to mild GSM (*p* < 0.001). This 7.6-point reduction represents an objective revision of the clinical signs of GSM at the vulvar level, consolidating the therapeutic efficacy of injectable collagen and surpassing the magnitude of effect reported by other regenerative therapies. Although the literature on the specific use of the vHI (Vulvar Health Index) in regenerative therapies such as PRP, hyaluronic acid or laser is limited, there are relevant studies using the VHI (Vaginal Health Index), a complementary index validated in the diagnosis of GSM, allowing indirect comparisons with our results. For example, Saleh and Abdelghani [24] reported a significant improvement in VHI after PRP injections in post-menopausal women, observing average increases of up to 6 points in one month. Similarly, Hersant et al. [39] evaluated the combination of PRP and hyaluronic acid in breast cancer survivors, with a mean increase in VHI from 10.7 ± 2.1 to 20.8 ± 4.8 at 6-month follow-up. Angelucci et al. [40] on the other hand, used hyaluronic acid injections with polynucleotides, obtaining statistically significant improvements in both VHI and vHI. In this context, the changes observed in our cohort, with increases of +8.9 points in the VHI and decreases of −7.6 points in the vHI, stand out for their magnitude and clinical consistency. These preliminary results suggest that collagen may be a promising regenerative alternative for GSM and highlight its potential as part of a broader therapeutic strategy; however, further controlled studies are needed to confirm these findings. To date, no studies have been published documenting the specific use of injectable collagen in GSM treatment. There are, however, precedents in other pelvic floor and musculoskeletal dysfunctions that support its potential regenerative effect. For example, the clinical trial by Romero-Cullerés et al. [34], in women with post-partum perineal scarring, applied scales such as the Vancouver Scar Scale (VSS) and the Patient Scar Assessment Scale (PSAS), which assess similar parameters to those considered in the GSM scales. The VSS, an objective clinical instrument, assesses four parameters: vascularity, pigmentation, pliability, and height, giving an overall score between 0 (normal skin) and 13 (worst scar imaginable). The PSAS, a self-administered questionnaire, consists of six items assessing pain, itchiness, color, hardness, thickness, and irregularity, each on a scale of 1 to 10, with the results added up to generate a subjective assessment from the patient’s perspective. In the study, both groups showed improvement in all four domains of the VSS, and patients in the collagen group perceived significant improvements in all six aspects assessed by the PSAS, reflecting visible, palpable, and symptomatic changes in scar structure and quality, to the extent that some scores approached normal skin values. These findings suggest that the parameters assessed by these scales might be conceptually applicable to the context of GSM, as indicators such as vascularity, elasticity, epithelial integrity, and absence of symptoms (e.g., itching, pain) are also relevant in the assessment of vulvovaginal trophism. However, further studies would be needed to confirm the validity of such extrapolation. Similarly, research has explored the biostimulatory and regenerative effect of injectable collagen in other pelvic floor dysfunctions, such as in the treatment of stress urinary incontinence. Leonhardt et al. [41] described the efficacy of cystoscopy-guided periurethral collagen injections assessed by transvaginal ultrasound, noting a good clinical response with no relevant complications. More recently, the study by Defreitas GA et al. [42] has provided an innovative approach using three-dimensional ultrasound as an objective tool to assess collagen distribution after periurethral injection. This work demonstrated that a homogeneous circumferential distribution of collagen was significantly associated with better clinical outcomes, underlining the importance of not only the injected material but also of its correct localization and tissue dispersion. Although the mechanisms of action differ, these examples share a common therapeutic goal: restoring function through direct intervention on damaged connective tissue, which reinforces the rationale behind their use in GSM. The biological effects of injectable collagen at the tissue level showed a positive impact on GSM symptomatology in our study. The results of the Vulvovaginal Symptoms Questionnaire (VSQ) indicated a significant improvement in 100% of participants, with marked decreases in itching, burning or stinging, pain, irritation, dryness, vaginal or vulvar secretion, worry about vulvar symptoms, worry about the appearance of the vulva, frustration surrounding symptoms, feeling embarrassed about symptoms, effects of symptoms on social interactions, affecting the desire to be with other people, difficulty showing affection due to symptoms, affecting daily activities, affecting the desire for intimacy, being sexually active with a partner, effect on sexual relations; pain, dryness, and bleeding during sexual relations. These results reflect not only objective improvements, but also a substantial improvement in quality of life. This finding is consistent with the literature on different regenerative therapies in GSM. For example, Saleh et al. [24] observed significant improvements in VSQ items, including burning, pain, irritation, dryness, vaginal discharge, sexual desire and dyspareunia, one month after two intravaginal platelet-rich plasma (PRP) injections in post-menopausal women with vulvovaginal atrophy (*p* = 0.045 for vaginal dryness and <0.001 for all other items). Reductions of 60–80% in symptoms such as dyspareunia and dryness were reported. Responses were, however, more heterogeneous than those obtained with collagen in our cohort. In our study, 100% of participants reported improvement in at least one domain of the VSQ, covering physical symptoms as well as emotional and functional impairments. On the other hand, the meta-analysis conducted by Moccia F et al. [18] on injectable therapies in the context of GSM showed improvements in VSQ scores with the use of PRP, both alone and in combination with hyaluronic acid or other regenerative biomaterials. Finally, a more recent pilot study by Oyardi et al. [35] evaluated the efficacy of platelet-rich fibrin (i-PRF), observing significant improvements in sexual quality and VSQ domains in a sample of 35 women. All these improvements, both symptomatic and tissue-related, were also reflected in the satisfaction levels reported by patients. According to the Clinical Global Impression Scale (CGI-I), 80% of women reported feeling ‘much better’ after treatment, while 20% reported ‘moderate’ improvement, indicating a generally positive perception of the effects of injectable collagen. This finding is particularly relevant when compared to previous studies of other regenerative injectable therapies in GSM. For example, in the prospective study by Berreni et al. [13], 95% of women treated with cross-linked hyaluronic acid (DESIRIAL^®^) reported some improvement in their vulvovaginal symptomatology, but only 40% reported feeling ‘much better’ according to the Patient Global Impression of Improvement Scale (PGI-I). This difference could be due to the specific biostimulatory properties of collagen, as well as its deeper tissue mechanism of action, which promotes functional restoration of the extracellular matrix and improves both the structure and sensitivity of GSM-affected tissues. These results are equally backed up by a recent systematic review [18], which evaluated eight studies with a total of 236 women undergoing regenerative injectable treatments for GSM (PRP, HA, CaHA, microfat + PRP, among others). Patient satisfaction, as measured by the Clinical Global Impression Scale (PGI-I/CGI-I), showed improvements ranging from 70% to 90% after regenerative therapies. In contrast, in our cohort, 80% of patients reported feeling ‘much better’ and 20% ‘moderately better’, which places our injectable collagen treatment in the upper range of satisfaction and even suggests a higher positive perception of therapeutic response.

These findings suggest a potential association between injectable collagen and symptomatic improvement, as well as patient satisfaction in our cohort; however, these observations and comparisons with other regenerative therapies (PRP or hyaluronic acid) should be interpreted with caution due to the study’s limitations. Regarding safety, no relevant adverse effects were recorded in our series (100%), suggesting a favorable tolerability profile of intradermal collagen in the treatment of GSM. The absence of complications may be related, at least in part, to the rigorous application of aseptic measures and adherence to post-procedure recommendations (abstinence from sexual intercourse for one week, application of localized cold on underwear, and avoidance of bathing in public pools). These findings are in line with those described in the literature for other regenerative therapies, which have reported low or no complication rates with PRP [35] those of Ragy et al. [36] who cross-linked hyaluronic acid and polynucleotides + HA [40], as well as in case series involving collagen injections in perineal scars [34]. Although these preliminary data support the potential safety of injectable collagen, further studies with larger cohorts and longer follow-up are needed to confirm these findings.

As previously mentioned, this study has important limitations that must be considered when interpreting the results. First, its retrospective design carries the risk of selection and information bias, as it relies on prior clinical records and the consistency of data collected in medical histories. Second, the small sample size (*n* = 20) limits statistical power, increases the uncertainty of effect estimates, and reduces the generalizability of the findings to other populations. Furthermore, the absence of a control group prevents attributing the observed improvements solely to collagen, as the influence of the natural course of GSM, placebo effects, concomitant behavioral changes, or the possible impact of adjunct measures (e.g., hygienic-dietary recommendations or changes in the use of lubricants/moisturizers) cannot be ruled out. Another limitation is the variability in exposure (total number of sessions) and the fact that the follow-up focused on a single time point (three months after the last injection), meaning the duration of the effect and the need for retreatments cannot be precisely established. Finally, although validated scales were used (VAS, VHI, vHI, VSQ, and CGI-I), most are clinical and self-reported measures, and the study did not incorporate biomarkers or histological confirmation to directly objectify tissue changes (epithelium, vascularization, extracellular matrix) or correlate them with clinical improvement.

Despite these limitations, the study provides a reflection of real clinical practice and highlights an important finding: the identification of GSM cases initially masked by other pelvic pain syndromes, emphasizing the importance of differential diagnosis and specific evaluation in pelvic floor units. Based on these preliminary results, a controlled prospective study has been initiated to more rigorously assess the efficacy and duration of the effect of intradermal collagen in GSM, with direct comparisons to other regenerative therapies (PRP, hyaluronic acid, or laser), a standardized definition of the number of sessions, and the incorporation of additional objective measures to better characterize the clinical and functional response to treatment. It is important to note that, due to the retrospective design, the pilot nature of the study, the absence of a control group, and the limited follow-up period, definitive causal inferences cannot be drawn. Therefore, the results should be interpreted with caution and considered exploratory and hypothesis-generating.

## 5. Conclusions

The efficacy of collagen injections in the treatment of genitourinary syndrome of menopause (GSM) was evaluated through the reduction in pain on the VAS and, secondarily, the improvement of vulvovaginal functional parameters. The results showed a significant decrease in pain, both at rest and during sexual intercourse, as well as a substantial improvement in vaginal and vulvar health indices, reaching values compatible with mild GSM or even clinical resolution of the syndrome. These preliminary findings suggest that injectable collagen could represent a promising therapeutic alternative for patients with GSM refractory to conventional treatment, given its apparent tolerability, safety, and potential sustained effect. However, the absence of a control group and the limited sample size are important limitations that prevent definitive conclusions. Therefore, these results should be considered exploratory and hypothesis-generating. Further studies with larger sample sizes and randomized controlled designs comparing this intervention with other standard treatments are required to confirm these findings and define its potential inclusion in future clinical guidelines.

## Figures and Tables

**Table 1:** Signs and Symptoms of GSM.

Genital	Sexual	Urinal
Vaginal dryness	Dyspareunia	Dysuria
Irritation, burning, itchiness	Decreased lubrication	Urgent urination
Leucorrhoea	Post-coital bleeding	Urethral prolapse
Thinning of the vaginal mucosa	Loss of libido and arousal	Stress/urgency urinary incontinence
Vaginal or pelvic pain	Dysorgasmia	Predisposition to urinary tract infections
Vaginal vault prolapse		Nocturia
Retraction/thinning of labia minora and labia majora		Polyuria
Retraction of the vaginal introitus		
Loss of pubic hair		
Vulvar pallor		
Disappearance of the clitoral hood		

**Table 2:** Exploratory analysis of demographic and clinical variables.

Variable	*n* = 20
Age	
Median (Range)	54 (48–58)
Mean (SD)	54 (8)
Reason for referral, *n* (%)	
Dyspareunia	1 (5.0%)
Pelvic pain	11 (55.0%)
Urinary incontinence	6 (30.0%)
Vulvodynia	2 (10.0%)
Main diagnosis (in rhb consultation), *n* (%)	
GSM	20 (100.0%)

**Table 3:** Exploratory analysis of prior GSM treatments.

Prior GSM Treatments	*n* = 20
Lubricant treatment (%)	
No	9 (45.0%)
Yes	11 (55.0%)
Moisturizing treatment (without hyaluronic acid) (%)	
No	15 (75.0%)
Yes	5 (25.0%)
Moisturizing treatment (hyaluronic acid) (%)	
No	10 (50.0%)
Yes	10 (50.0%)
Local estrogen therapy (%)	
No	16 (80.0%)
Yes	4 (20.0%)
Systemic estrogen therapy (%)	
No	19 (95.0%)
Yes	1 (5.0%)
Ospemifene (%)	
No	19 (95.0%)
Yes	1 (5.0%)
Lidocaine cream (%)	
No	19 (95.0%)
Yes	1 (5.0%)

**Table 4:** Exploratory analysis of the most common signs and symptoms.

Most Common Signs and Symptoms	*n* = 20
Dyspareunia/sexual dysfunction (%)	
No	5 (25.0%)
Yes	15 (75.0%)
Dryness (%)	
No	2 (10.0%)
Yes	18 (90.0%)
Irritation/Itching/Burning (%)	
Yes	20 (100.0%)
Over-active bladder (%)	
No	19 (95.0%)
Yes	1 (5.0%)
Urinary incontinence (%)	
No	11 (55.0%)
Yes	9 (45.0%)

**Table 5:** Pre- and post-intervention comparison of VSQ.

Variable	Overall *n* = 40	PRE *n* = 20	POST *n* = 20	*p*-Value
Q1 Have you experienced vulvar itching?				<0.001
No	20 (50%)	0 (0%)	20 (100%)	
Yes	20 (50%)	20 (100%)	0 (0%)	
Q2 Do you feel your vulva burning or stinging?				<0.001
No	20 (50%)	0 (0%)	20 (100%)	
Yes	20 (50%)	20 (100%)	0 (0%)	
Q3 Are you experiencing vulva pain?				<0.001
No	16 (40%)	0 (0%)	16 (80%)	
Yes	24 (60%)	20 (100%)	4 (20%)	
Q4 Is you vulva irritated?				<0.001
No	20 (50%)	0 (0%)	20 (100%)	
Yes	20 (50%)	20 (100%)	0 (0%)	
Q5 Do you have vaginal or vulva dryness?				<0.001
No	21 (53%)	2 (10%)	19 (95%)	
Yes	19 (48%)	18 (90%)	1 (5.0%)	
Q6 Are you experiencing any discharge from your vulva or vagina?				0.005
No	31 (78%)	19 (95%)	12 (60%)	
Yes	9 (23%)	1 (5.0%)	8 (40%)	
Q7 Do you notice any oduor from your vulva or vagina?				0.5
No	37 (93%)	18 (90%)	19 (95%)	
Yes	3 (7.5%)	2 (10%)	1 (5.0%)	
Q8 Do you worry about your vulvar symptoms?				<0.001
No	20 (50%)	1 (5.0%)	19 (95%)	
Yes	20 (50%)	19 (95%)	1 (5.0%)	
Q9 How do you feel about the appearance of your vulva?				<0.001
No	20 (50%)	1 (5.0%)	19 (95%)	
Yes	20 (50%)	19 (95%)	1 (5.0%)	
Q10 Do you feel frustrated about your vulvar symptoms?				<0.001
No	20 (50%)	0 (0%)	20 (100%)	
Yes	20 (50%)	20 (100%)	0 (0%)	
Q11 Are you embarrassed about your vulvar symptoms?				<0.001
No	22 (55%)	2 (10%)	20 (100%)	
Yes	18 (45%)	18 (90%)	0 (0%)	
Q12 How have your vulvar symptoms affected your interactions with others?				<0.001
No	24 (60%)	4 (20%)	20 (100%)	
Yes	16 (40%)	16 (80%)	0 (0%)	
Q13 Do your vulvar symptoms effect your desire to be with people?				<0.001
No	25 (63%)	5 (25%)	20 (100%)	
Yes	15 (38%)	15 (75%)	0 (0%)	
Q14 Have your vulvar symptoms made it hard to show affection?				<0.001
No	25 (63%)	5 (25%)	20 (100%)	
Yes	15 (38%)	15 (75%)	0 (0%)	
Q15 How do your vulvar symptoms affect your daily activities?				<0.001
No	22 (55%)	2 (10%)	20 (100%)	
Yes	18 (45%)	18 (90%)	0 (0%)	
Q16 Do your vulvar symptoms affect your desire to be intimate?				<0.001
No	20 (50%)	0 (0%)	20 (100%)	
Yes	20 (50%)	20 (100%)	0 (0%)	
Q17 Are you currently sexually active with a partner?				0.001
No	18 (45%)	14 (70%)	4 (20%)	
Yes	22 (55%)	6 (30%)	16 (80%)	
Q18 How have your vulvar symptoms affected your sexual relationships?				<0.001
No	20 (50%)	0 (0%)	20 (100%)	
Yes	20 (50%)	20 (100%)	0 (0%)	
Q19 Do your vulvar symptoms cause pain during sexual activity?				<0.001
No	14 (35%)	0 (0%)	14 (70%)	
Yes	26 (65%)	20 (100%)	6 (30%)	
Q20 Do your vulvar symptoms cause dryness during sexual activity?				<0.001
No	20 (50%)	0 (0%)	20 (100%)	
Yes	20 (50%)	20 (100%)	0 (0%)	
Q21 Do your vulvar symptoms cause bleeding during sexual activity?				<0.001
No	20 (50%)	0 (0%)	20 (100%)	
Yes	20 (50%)	20 (100%)	0 (0%)	

**Table 6:** Results of Clinical Global Impression Scale.

Variable	*n* = 20
CGI, *n* (%)	
Moderately better	4 (20%)
Much better	16 (80%)

## Data Availability

The data that support the findings of this study are available from the corresponding author upon reasonable request.

## Supplementary Materials

The following supporting information can be downloaded at https://www.mdpi.com/article/10.3390/jcm15041408/s1, Figure S1: VAS Figure; Table S1: VHI table; Table S2: vHi table; Table S3: VSQ table; Table S4: CGI table.
