# Supplementary material for: Vulvovaginal Collagen Injection as a Regenerative Strategy in Genitourinary Syndrome of Menopause: Results of a Pilot Study
Source: J Clin Med. 2026 Feb 11;15(4):1408. doi: 10.3390/jcm15041408 (PMC12942073; doi:10.3390/jcm15041408)
Supplement: Supplementary file 1 [file jcm-15-01408-s001.zip › jcm-4090549-supplementary.pdf]

Figure S1.

Visual analogue scale VAS

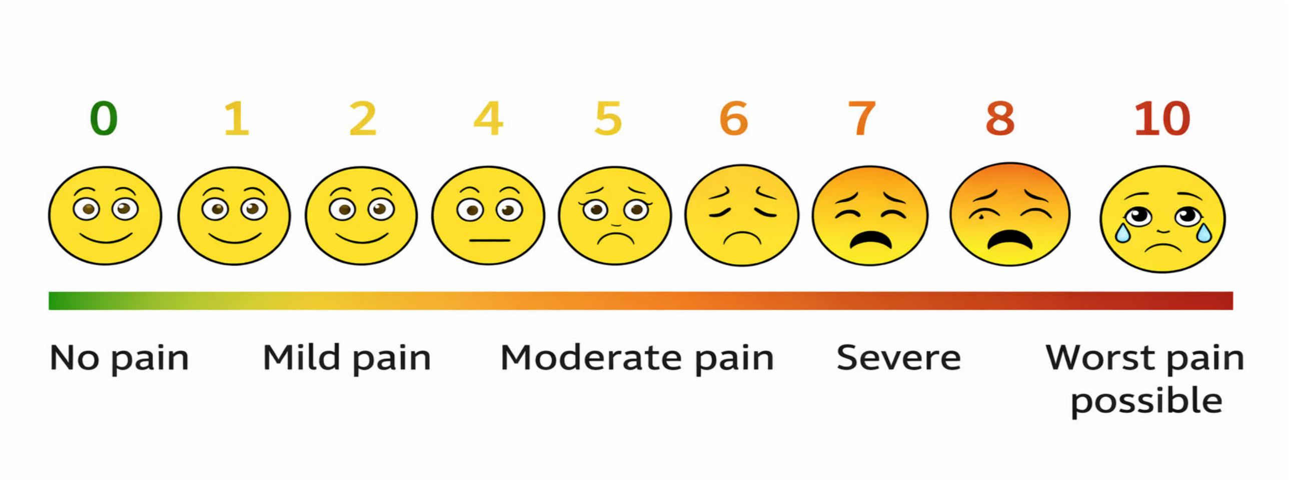

Table S1

Modified Vaginal Health Index

Score	1	2	3	4	5
Elasticity	None	Poor	Acceptable	Good	Excellent
Discharge volume	None	Poor	Thin layer on the surface	Moderate layer	Normal
Integrity of the epithelium	Spontaneous petechiae	Bleeding at the slightest touch	Bleeding on scratching	Epithelium not prone to bleeding	Normal
Moisture	None, inflamed surface	None, normal surface	Minimal	Moderate	Normal

Scores below 12 correspond to atrophy.

Table S2
Vulvar health index

	Normal (0)	Mild (1)	Moderate (2)	Severe (3)
Labia majora and labia minora	Normal	Slight loss	Moderate loss	Severe loss or disappearance
Clitoris	Normal size	Slight decrease in size	Moderate decrease in size	Severe decrease in size or undetectable
Vestibule and elasticity	Normal	Mild decrease or narrowing	Moderate decrease or narrowing	Severe decrease or narrowing
Colour	Normal	Mild pallor	Moderate pallor	Severe pallor
Discomfort and pain	No	Mild during intercourse	Moderate during intercourse	Severe during intercourse

A score of 0-5 corresponds to mild atrophy; 5-10 moderate and >10 atrophy is considered severe.

Table S3
Vulvovaginal Symptoms Questionnaire

During the last week, have you been bothered by:	Yes	No
Have you experienced vulvar itching?		
Do you feel your vulva burning or stinging?		
Are you experiencing vulva pain?		
Is your vulva irritated?		
Do you have vaginal or vulva dryness?		
Are you experiencing any discharge from your vulva or vagina?		
Do you notice any odour from your vulva or vagina?		
Do you worry about your vulvar symptoms?		
How do you feel about the appearance of your vulva?		
Do you feel frustrated about your vulvar symptoms?		
Are you embarrassed about your vulvar symptoms?		
How have your vulvar symptoms affected your interactions with others?		
Do your vulvar symptoms effect your desire to be with people?		
Have your vulvar symptoms made it hard to show affection?		
How do your vulvar symptoms affect your daily activities?		
Do your vulvar symptoms affect your desire to be intimate?		
Are you currently sexually active with a partner?		
How have your vulvar symptoms affected your sexual relationships?		
Do your vulvar symptoms cause pain during sexual activity?		
Do your vulvar symptoms cause dryness during sexual activity?		
Do your vulvar symptoms cause bleeding during sexual activity?		

Table S4
Clinical Global Impression scale

**Rate the relief obtained from the treatment**	
Far better	1
Much better	2
A little better	3
No change	4
A little worse	5
Much worse	6
Far worse	7
